# Neural mechanisms of negative reinforcement in children and adolescents with autism spectrum disorders

**DOI:** 10.1186/s11689-015-9107-8

**Published:** 2015-03-25

**Authors:** Cara R Damiano, Dillon C Cockrell, Kaitlyn Dunlap, Eleanor K Hanna, Stephanie Miller, Joshua Bizzell, Megan Kovac, Lauren Turner-Brown, John Sideris, Jessica Kinard, Gabriel S Dichter

**Affiliations:** Department of Psychology, University of North Carolina at Chapel Hill, CB #3270, Davie Hall, 27599-3270 Chapel Hill, NC USA; Carolina Institute for Developmental Disabilities, University of North Carolina at Chapel Hill School of Medicine, CB #7255, 27599-7255 Chapel Hill, NC USA; Virginia Tech Carilion School of Medicine and Research Institute, 2 Riverside Circle Suite M140, 24016 Roanoke, VA USA; Brain Imaging and Analysis Center (BIAC), Duke University Medical Center, 2424 Erwin Road Suite 501, 27708 Durham, NC USA; Center for Cognitive Neuroscience, Duke University, Box # 90999, 27708 Durham, NC USA; UNC School of Social Work, University of North Carolina at Chapel Hill School of Education, CB #3550, 27599-3550 Chapel Hill, NC USA; Department of Psychiatry, University of North Carolina at Chapel Hill School of Medicine, CB #7160, 27599-7160 Chapel Hill, NC USA; School Psychology Program, University of North Carolina at Chapel Hill School of Education, CB #3550, 27599-3550 Chapel Hill, NC USA; TEACCH Autism Program, CB# 7180 UNC-Chapel Hill, 27599-7180 Chapel Hill, NC USA; Frank Porter Graham Child Development Institute, University of North Carolina at Chapel Hill, CB #8185, 27599-8185 Chapel Hill, NC USA

**Keywords:** Autism spectrum disorders (ASD), Negative reinforcement, Reward processing, Social motivation, Reward loss, Reward motivation

## Abstract

**Background:**

Previous research has found accumulating evidence for atypical reward processing in autism spectrum disorders (ASD), particularly in the context of social rewards. Yet, this line of research has focused largely on positive social reinforcement, while little is known about the processing of negative reinforcement in individuals with ASD.

**Methods:**

The present study examined neural responses to social negative reinforcement (a face displaying negative affect) and non-social negative reinforcement (monetary loss) in children with ASD relative to typically developing children, using functional magnetic resonance imaging (fMRI).

**Results:**

We found that children with ASD demonstrated hypoactivation of the right caudate nucleus while anticipating non-social negative reinforcement and hypoactivation of a network of frontostriatal regions (including the nucleus accumbens, caudate nucleus, and putamen) while anticipating social negative reinforcement. In addition, activation of the right caudate nucleus during non-social negative reinforcement was associated with individual differences in social motivation.

**Conclusions:**

These results suggest that atypical responding to negative reinforcement in children with ASD may contribute to social motivational deficits in this population.

## Background

### Neural mechanisms of negative reinforcement in autism spectrum disorders

Autism spectrum disorders (ASD) are characterized by deficits in social interaction and communication as well as restricted and repetitive behaviors and interests [1]. It has been suggested that core social symptoms in ASD may be related to impaired social motivation [2,3]. The ‘social motivation hypothesis’ of ASD posits that atypical reward motivation may lead to hyporesponsivity to social rewards, which in turn causes individuals with ASD to forgo key opportunities for engaging in social learning throughout development. Consistent with this model, children with ASD report reduced responsivity to social rewards [4,5] and demonstrate reduced attention towards social stimuli relative to non-social stimuli [6]. In recent years, neuroimaging research has further substantiated the social motivation hypothesis of ASD by providing evidence for reward system dysfunction in ASD (see [7], for a review). Collectively, these studies implicate a circumscribed network of reward processing regions, including primarily prefrontal cortical and striatal regions, that function atypically in individuals with ASD, with the most consistent finding being attenuated striatal activation during the anticipation or receipt of monetary and social rewards [8-11].

However, despite the growing body of literature addressing reward processing deficits in ASD, most studies to date have focused only on responses to positive reinforcement, whereas very little is known about the processing of negative reinforcement in ASD. Positive reinforcement refers to a stimulus (typically positive reward gain) that motivates approach-related behaviors while negative reinforcement refers to a stimulus that facilitates behaviors to avoid loss or punishment [12]. Only two studies to date have previously addressed negative reinforcement in ASD. In a risk-taking task, South and colleagues [13] found that children and adolescents with ASD showed different physiological responses when anticipating possible non-social negative reinforcement and reported evidence that individuals with ASD and high levels of anxiety may be particularly motivated to avoid negative reinforcement. In another study examining electrophysiological responses to non-social negative reinforcement in ASD, no group differences were found for feedback-related negativity amplitude, but the amplitude of this ERP component was correlated with age in the ASD group, as younger children with ASD demonstrated more attenuated responses to negative reinforcement [14].

However, there are no published studies on the neural correlates of negative reinforcement in ASD, which is notable, given that mesolimbic reward circuitry evolved to guide behavior in the context of both social and non-social and positive and negative reinforcement [15,16]. Preclinical research suggests that mesolimbic dopamine neurons fire in response to the anticipation of positive *and* negative reinforcers [17,18], and functional neuroimaging studies in human subjects indicate that negative reinforcement is associated with a similar functional neuroanatomy as positive reinforcement [19,20], including activation of the ventral and dorsal striatum, insula, orbital frontal cortex (OFC), and anterior cingulate cortex.

The striatum in particular is responsive to the anticipation of both positive and negative reinforcement. For instance, the anticipation and experience of monetary reward loss, or other aversive stimuli (such as, electric shocks), are associated with altered activity of the nucleus accumbens (NAc) [21-24]. The dorsal striatum (which includes the caudate nucleus and putamen) is also associated with the anticipation and processing of aversive stimuli [23,25-28] and plays a critical role in altering future behavior following negative reinforcers [29,30]. Negative reinforcement is also associated with activation of the insula [27,31,32], which is active during the anticipation of emotionally aversive stimuli [33] and during decisions involving risk [34]. The OFC has also been associated with the processing of negative reinforcement [35], which may reflect the role of the OFC in encoding information about reward expectation [36-38] and in modulating dopaminergic neurons that convey information about expected reward value [39]. Negative reinforcement has also been linked to activation of the anterior cingulate cortex [28], which is associated with integrating information about reward prediction and outcome [40,41], as well as the amygdala [23,42], which is activated during emotionally salient events [43] and reward learning [44].

In the context of social rewards, negative reinforcement is particularly important because social motivation is comprised of both the drive to pursue social rewards (for example, seeking out novel friendships) *and* sensitivity to potential negative social outcomes (for example, changing behavior in response to unreciprocated social bids) [45]. Since negative reinforcement (such as, social rejection or disapproval) is known to exert a strong influence on social behavior [45,46], impaired social motivation in ASD may reflect either a reduced drive for social rewards or a diminished responsivity to potential social punishment. Although there is currently little research on the neural correlates of negative social reinforcement, a recent study found that the anticipation of avoidable social punishment (that is, videos depicting social disapproval) was associated with activation of the NAc [46].

The objective of the present study was to examine neural responses to social and non-social negative reinforcement in children with ASD. We used an adapted version of the monetary incentive delay (MID) task [28] that included runs in which participants anticipated avoidable monetary loss or sad faces. We conceptualized these conditions as reflecting non-social and social negative reinforcement, respectively, because participants responded with speeded button presses to avoid these negative outcomes that in turn would serve to influence behavior on subsequent trials. Our hypothesis was that children with ASD would demonstrate reduced activation of mesolimbic reward circuitry in response to negative social reinforcement in frontostriatal reward processing regions. We also hypothesized that the magnitude of neural activation in frontostriatal regions during the anticipation and processing of negative social reinforcement would be associated with the severity of ASD symptoms in the ASD group.

## Methods

### Participants

The original sample for this study included 22 typically developing children and 26 children with ASD ranging in age from 9 to 18 years old. From this sample, one typically developing child and two children with ASD were excluded from analyses due to falling asleep during the scan session and/or excessive head motion (>5 mm along any of the six possible axes, including *x*, *y*, *z*, pitch, yaw, and roll). In addition, four participants with ASD (two participants per run type) demonstrated excessive movement in one of the two runs, and thus, these runs were selectively dropped from analyses. The final sample included 21 typically developing children (17 males, 4 females) and 24 children with ASD (23 males, 1 female). No significant differences were found between groups in terms of age, sex, intelligence quotient (IQ) scores (full-scale, verbal, or non-verbal), or race/ethnicity, all *P*’s > .05 (see Table 1; IQ scores were measured by the Kaufman Brief Intelligence Test (KBIT) [47]). Thirteen children in the ASD group were on psychotropic medication, including psychostimulants (Vyvanse, Adderall, Focalin), atypical antidepressants (Bupropion), antihypertensives/central alpha-2 adrenergic agonists (Tenex, Clonidine, Intuniv), benzodiazepines (Klonopin), selective serotonin reuptake inhibitors (SSRIs) (Prozac, Zoloft), mood stabilizers (Depakote), and atypical antipsychotics (Risperdal, Abilify). All ASD participants had clinical diagnoses of ASD that were confirmed through the Autism Diagnostic Observation Schedule (ADOS) [48] administered by trained research staff supervised by a licensed clinical psychologist and using standard cutoffs. Because both Modules 3 and 4 were used (Module 3: 11 participants, Module 4: 13 participants), calibrated severity scores were calculated from raw scores ADOS scores to obtain a dimensional measure of ASD symptom severity across both Modules (see Table 1) [49,50]. All participants gave informed consent to participate in the present study. The informed consent form and study protocol were approved by the Institutional Review Board of Duke University.

**Table 1:** **Means and standard deviations (SD) for the ASD and control groups**

	**Control (** ***N*** **= 21) Mean (SD)**	**ASD (** ***N*** **= 24) Mean (SD)**
Age	14.25 (2.98)	14.35 (3.16)
Full scale IQ^a^	110.79 (13.52)	106.96 (16.35)
Verbal IQ^a^	110.16 (14.25)	105.71 (17.95)
Performance IQ^a^	108.47 (12.09)	106.00 (15.40)
SRS^b^		
Awareness	10.79 (2.27)	9.04 (2.91)
Cognition	11.16 (2.27)*	16.21 (5.35)*
Communication	17.63 (3.29)*	25.38 (6.92)*
Mannerisms	2.32 (2.24)*	15.92 (5.30)*
Social motivation	9.58 (2.92)*	13.38 (3.61)*
Total score	51.47 (3.34)*	79.92 (18.46)*
Mean absolute head motion^c^	0.045 (0.09)	0.058 (0.10)
ADOS calibrated severity score^d^	-	8.25
Sex	17 males, 4 females	23 males, 1 female
Race	15 Caucasians, 6 African-Americans	22 Caucasians, 2 African-Americans

^a^IQ refers to the intelligence quotient derived from the Kaufman Brief Intelligence Test (KBIT); ^b^SRS refers to the social responsiveness scale [61]; ^c^Average head displacement in millimeter across six planes (x, y, z, pitch, yaw, and roll); ^d^Standardized severity scores on a scale of 1 to 10 calculated from raw Autism Diagnostic Observation Schedule (ADOS) scores [49,50]. *P < .05.

### fMRI task

**Figure 1:**
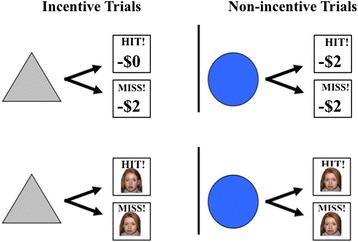
**The fMRI incentive delay task.** On alternative runs, participants made speeded button responses to avoid a monetary loss or avoid seeing an image of a sad face. The triangle cue indicated the opportunity to avoid losing money or seeing a sad face. The circle cue indicated that money would be lost or a sad face would be seen regardless of the speed of response.

The functional magnetic resonance imaging (fMRI) task was modified from a reward loss version of the monetary incentive delay (MID) task as implemented in Knutson *et al*. [28]. In this task, participants complete speeded button presses to avoid reward losses. All runs included both *incentive trials* (in which participants had the opportunity to avoid losses with adequately quick responses to a target) and *non-incentive trials* (in which participants were not able to avoid reward losses yet were still required to complete a speeded button press). Potential incentive and non-incentive trials were aperiodic and pseudorandomly ordered. Task conditions and trial timings are summarized in Figure 1. Each trial consisted of (1) a 2,000-ms cue indicating whether the trial was an incentive trial (a triangle) or a non-incentive trial (a circle); (2) a 2,000- to 2,500-ms crosshair fixation; (3) a target bull’s-eye presented for up to 500 ms that signaled participants to complete a speeded button press; (4) 3,000 ms of feedback that indicated whether the trial was successful or not; and (5) a variable length ITI crosshair resulting in a total trial duration of 12 s. Each 8-min run contained 40 trials, of which half were incentive trials. Run types (non-social, or monetary, runs and social, or face, runs) were presented in alternating and counter-balanced order. The task was adaptive such that participants were successful on two thirds of trials, regardless of individual differences in reaction times. Scanner stimuli were presented using E-Prime version 1.1 (Psychology Software Tools Inc., Pittsburgh, PA, USA) and displayed through magnet-compatible goggles (Resonance Technology, Inc., Northridge CA, USA).

As part of an hour-long scan session, participants completed two runs of this task (one social run and one non-social run). Each run type was indicated at the beginning of the run via an instruction screen and a verbal prompt from the experimenter. In the non-social, or monetary, version of the task, participants were provided with $72 at the start of the task and were instructed to try to lose as little money as possible, since they would receive whatever remained at the conclusion of the scan session. On incentive trials of the non-social run, participants were asked to respond to targets as quickly as possible to avoid losing $2 per trial. On non-incentive trials of the non-social run, participants also completed speeded button presses yet lost $2 regardless of the outcome. On both incentive and non-incentive trials, the feedback screen included an indication of whether participants had achieved an adequately quick response by displaying either ‘HIT!’ or ‘MISS!,’ as well as the amount that was lost or not lost on that trial through the display of either ‘−$0.00’ or ‘−$2.00’. At the bottom of the feedback screen, participants were informed of the total amount of money remaining based on their performance during the run thus far.

In the social version of the task, incentive trials allowed participants the chance to avoid seeing an image of a face with a sad expression and participants were instructed to avoid seeing sad faces as much as possible throughout the run. Following an adequately quick button response, participants were shown a face with a neutral expression instead of a sad face. In non-incentive trials of the social run, participants saw a face with a sad expression regardless of the speed of the response. Face stimuli included static closed mouth NimStim images with neutral and sad expressions [51]. As in the monetary condition, the feedback screen included an indication of whether the response was adequately quick (through displaying ‘HIT!’ or ‘MISS!’), as well as the total number of neutral or sad faces presented in the run thus far.

### Imaging methods

Scanning was performed on a General Electric (Wakuesha, WI, USA) 3.0 Tesla Signa Excite HD scanner. This scanner is equipped with high-power, high-duty-cycle 50-mT/m gradients at 200 T/m/s slew rate and 32-channel head coil. Head movement was restricted using foam cushions. An eight-channel head coil was used for parallel imaging. High-resolution T1-weighted structural images were acquired using a 3D FSPGR BRAVO pulse sequence (TR: 7.584 ms; Te: 2.936 ms; FOV: 25.6 cm; image matrix: 256^2^; voxel size: 1 × 1× 1 mm) and used for coregistration with the functional data. Structural images were aligned in the near-axial plane defined by the anterior and posterior commissures. Functional whole-brain images were acquired using a spiral pulse sequence with sensitivity-encoded (SENSE) parallel imaging reconstruction sensitive to blood oxygenation level-dependent (BOLD) contrast (TR: 1,500 ms; TE: 30 ms; FOV: 24 cm; isotropic voxel size: 3.75 × 3.75 × 4 mm). Runs began with four discarded RF excitations to allow for steady state equilibrium.

### Imaging data analysis

Functional data were preprocessed using FSL version 4.1.4 (Oxford Centre for Functional Magnetic Resonance Imaging of the Brain (FMRIB), Oxford University, UK). Preprocessing included the following steps: (1) brain extraction [52], (2) motion correction using MCFLIRT [53], (3) spatial smoothing using a Gaussian kernel of FWHM 5 mm, (4) mean-based intensity normalization of all volumes by the same factor, and (5) high-pass filtering. Functional and structural images were coregistered in native space and normalized to a standard stereotaxic space (Montreal Neurological Institute). Registrations were applied using an intermodal registration tool [53,54], and voxel-wise temporal autocorrelation was estimated and corrected using FMRIB’s improved linear model [55].

We note that we used a standard adult template brain for normalization of fMRI data although our sample included 9- to 18-year-old children. Normalization methods using linear scaling to standardize pediatric brains to an adult template result in negligible or relatively minor distortions in fMRI data for children older than 5 years of age [56]. While this approach may introduce some bias for studies involving high-resolution structural images, previous research demonstrates no limitations in using this method for functional images as in the present study [57]. In addition, the use of an adult template is common practice in studies with similar age ranges that include both ASD and typically developing (TD) participants [10,58,59].

Event onset times were used to model signal responses containing a regressor for each response type convolved with a double-γ function to model the hemodynamic response. Model fitting generated the whole-brain images of parameter estimates and variances, representing average signal changes from baseline. Group-wise activation images were calculated by a mixed effects higher level analysis using Bayesian estimation techniques, FMRIB Local Analysis of Mixed Effects [60] with cluster mean threshold of *Z* > 3.0 and a cluster-corrected significance threshold of *P* < 0.05 (FLAME 1 + 2) [61]. Anticipation and outcome phases were analyzed separately; within each, group differences with respect to responses to money and faces were modeled. Localizations were based on Harvard-Oxford cortical and subcortical structural probabilistic atlases as implemented in FSLView v3.0.

Whole-brain analyses were supplemented by follow-up analyses using anatomically defined regions of interest (ROIs) conducted to confirm the significant findings during the anticipation phase (see ‘Results’ section) in key striatal regions (right/left NAc, right/left caudate nucleus, and right/left putamen). These ROIs were defined using the Harvard-Oxford subcortical structural probabilistic atlas, thresholded at 25%, and binarized. For each ROI, each participant’s condition-specific mean parameter estimates were calculated using custom MATLAB scripts, and separate parameter estimates were calculated for the social and non-social conditions. These parameter estimates were then entered into a 2 (Group: ASD, TD) × 2 (Run Type: Social, Non-social) ANOVA.

Finally, correlations were evaluated between structural ROIs in the ventral and dorsal striatum (right/left NAc, right/left caudate nucleus, and right/left putamen) and scores on the Social Responsiveness Scale (SRS) [62], a self-report continuous measure of ASD symptom severity, for the ASD group. We examined these correlations using total *t*-scores from the SRS, as well as the social motivation subscale score, which measures individual differences in the drive to engage in interpersonal interactions [62] and which has previously been used to index social motivation in children and adolescents with ASD (for example, see [63,64]). Given the exploratory nature of these correlations, these analyses were not corrected for multiple comparisons.

## Results

### Mean absolute head displacement

After the exclusion of participants and runs with excessive motion (>5 mm along any of the six possible axes, including x, y, z, pitch, yaw, and roll), no significant differences were found between groups for mean absolute head motion along any of the six possible axes, all *P*’s > .05 (see Table 1).

### MID response times

**Figure 2:**
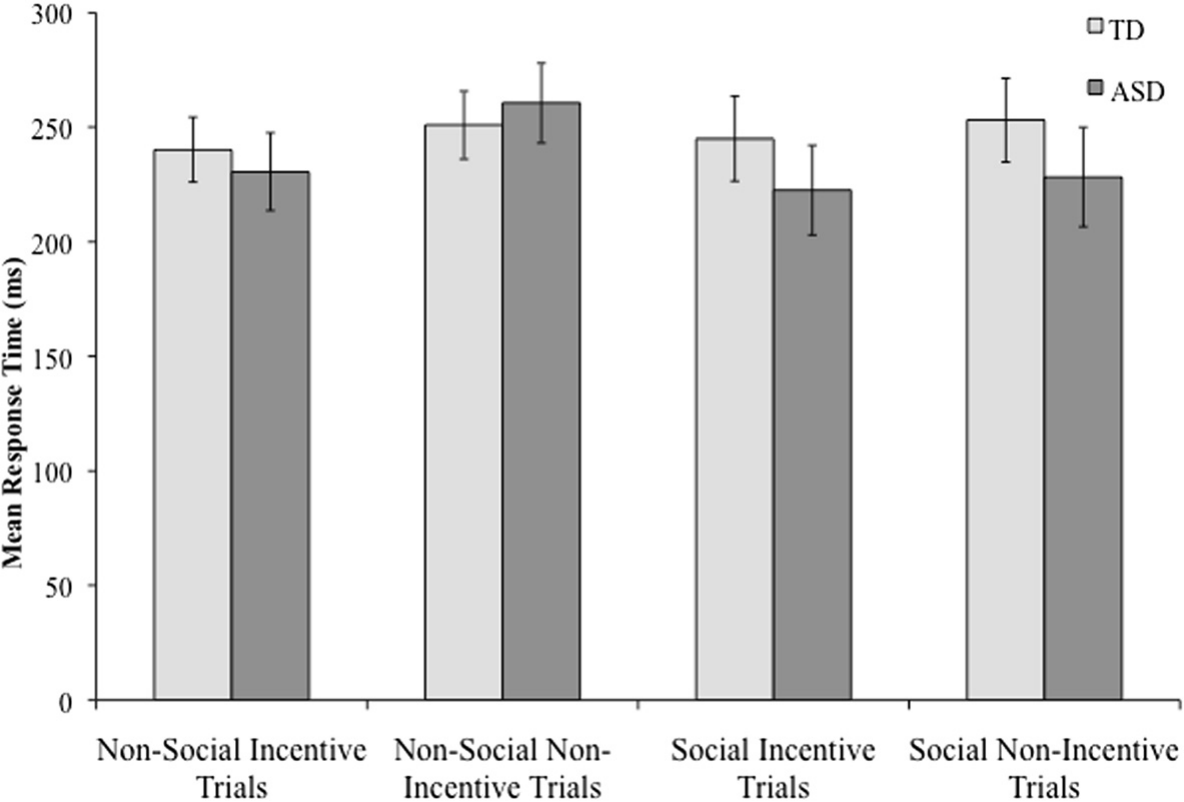
**Mean response times during the fMRI task.** Mean response times for incentive and non-incentive trials in the social and non-social conditions for the ASD and TD groups.

No differences were found between groups for the mean response times during the MID task in either the social or non-social runs, all *P*’s > .05 (see Figure 2).

### Functional whole-brain analyses

#### Anticipation phase: non-social condition

**Figure 3:**
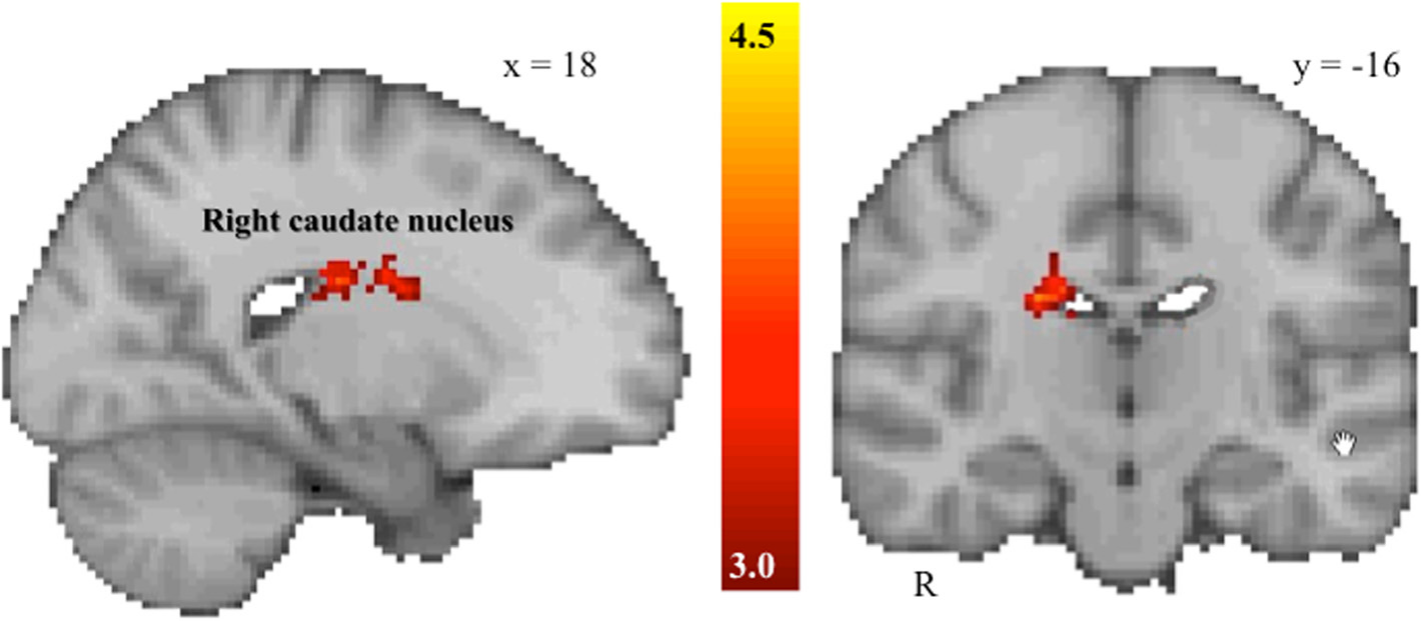
**Between-group differences in the non-social negative reinforcement condition.** The ASD group demonstrated significantly reduced activation in the right caudate nucleus during the anticipation of monetary loss, *Z* > 3.0, cluster-corrected *P* < .05, minimum cluster size = 10 voxels.

Anticipation of monetary loss was associated with significantly reduced activation in the right caudate nucleus in the ASD group relative to the TD group (see Table 2 and Figure 3). The ASD group did not demonstrate any clusters with significantly greater activation during the anticipation of monetary loss relative to the TD group.

**Table 2:** **Between-group differences in the non-social negative reinforcement condition**

			**MNI coordinates**		
**Brain region**	**Voxels**	***Z*** **(max)**	***X***	***Y***	***Z***
R caudate nucleus	200	4.22	16	−6	26

All coordinates (*x*, *y*, *z*) are given in MNI space. R, right. Clusters with decreased activation in the ASD group relative to the control group for the anticipation of monetary loss (thresholded at *Z* > 3.0, cluster-corrected significance threshold of *P* < .05).

#### Anticipation phase: social condition

**Figure 4:**
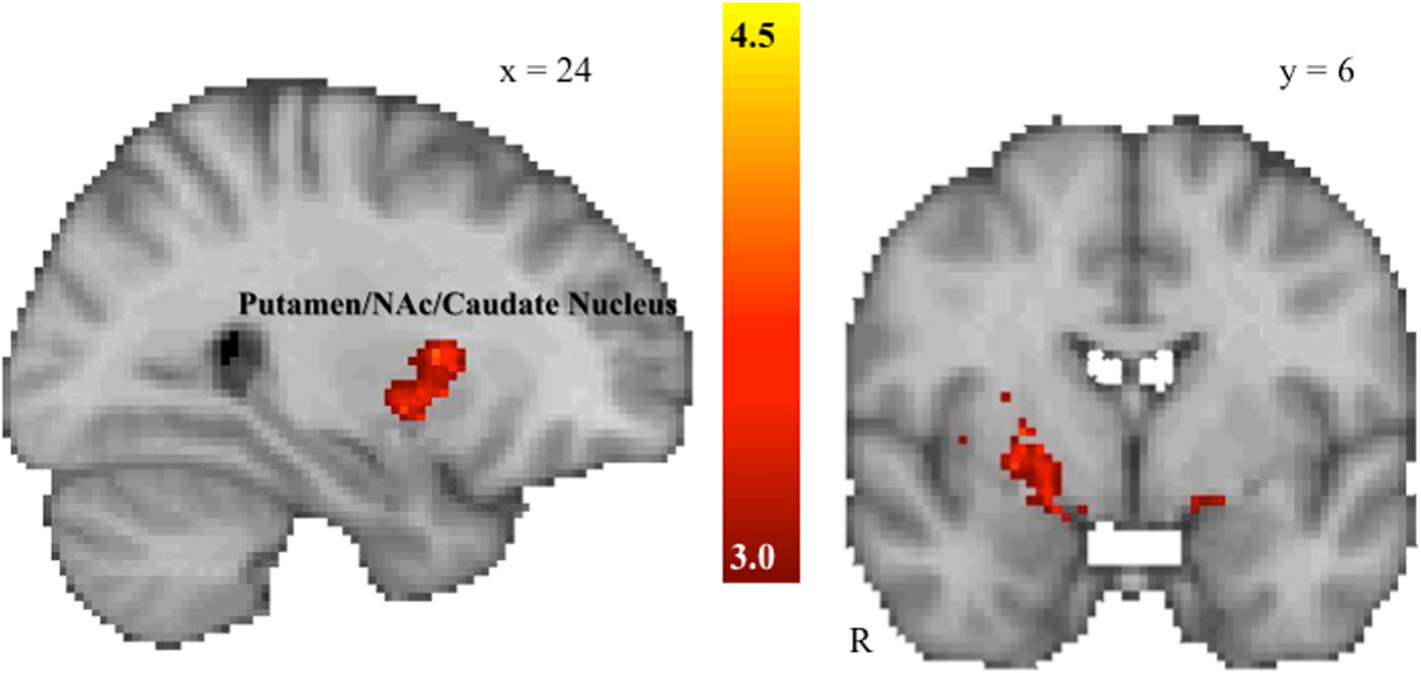
**Between-group differences in the social negative reinforcement condition.** The ASD group demonstrated significantly reduced activation in frontostriatal regions during the anticipation of sad faces, including a right-lateralized putamen/nucleus accumbens/caudate nucleus cluster, cluster-corrected *P* < .05, *Z* > 3.0, minimum cluster size = 10 voxels.

Anticipation of sad faces was associated with relatively reduced activation in two clusters spanning the right putamen/nucleus accumbens, left amygdala, bilateral insula, right frontal pole, right inferior frontal gyrus, and right anterior cingulate cortex in the ASD group compared to the TD group (see Table 3 and Figure 4). No clusters were significantly more activated in the ASD than in the TD group.

**Table 3:** **Between-group differences in the social negative reinforcement condition**

			**MNI coordinates**		
**Brain region**	**Voxels**	***Z*** **(max)**	***X***	***Y***	***Z***
L amygdala/putamen	29	4.57	20	−6	−12
R Anterior cingulate cortex^a^	21	3.66	8	22	30
R frontal pole	101	4.25	42	50	4
R inferior frontal gyrus, pars opercularis	23	3.98	60	14	6
L insula	26	4.51	−34	14	−2
R insula/orbital frontal cortex^a^	99	4.67	38	20	2
R putamen/nucleus accumbens/caudate nucleus	248	5.26	24	6	6

^a^Two clusters within same region, coordinates, and peak activation reported for highest peak activation. All coordinates (*x*, *y*, *z*) are given in MNI space. L, left, R, right. Clusters with decreased activation in the ASD group relative to the control group for the anticipation of sad faces (thresholded at *Z* > 3.0, cluster-corrected significance threshold of *P* < .05).

#### Outcome phase: monetary condition

No differences in brain activation were found between the ASD and TD groups during monetary reward loss outcomes.

#### Outcome phase: social condition

No differences in brain activation were found between the ASD and TD groups during sad face outcomes.

### Structural ROI analysis

**Figure 5:**
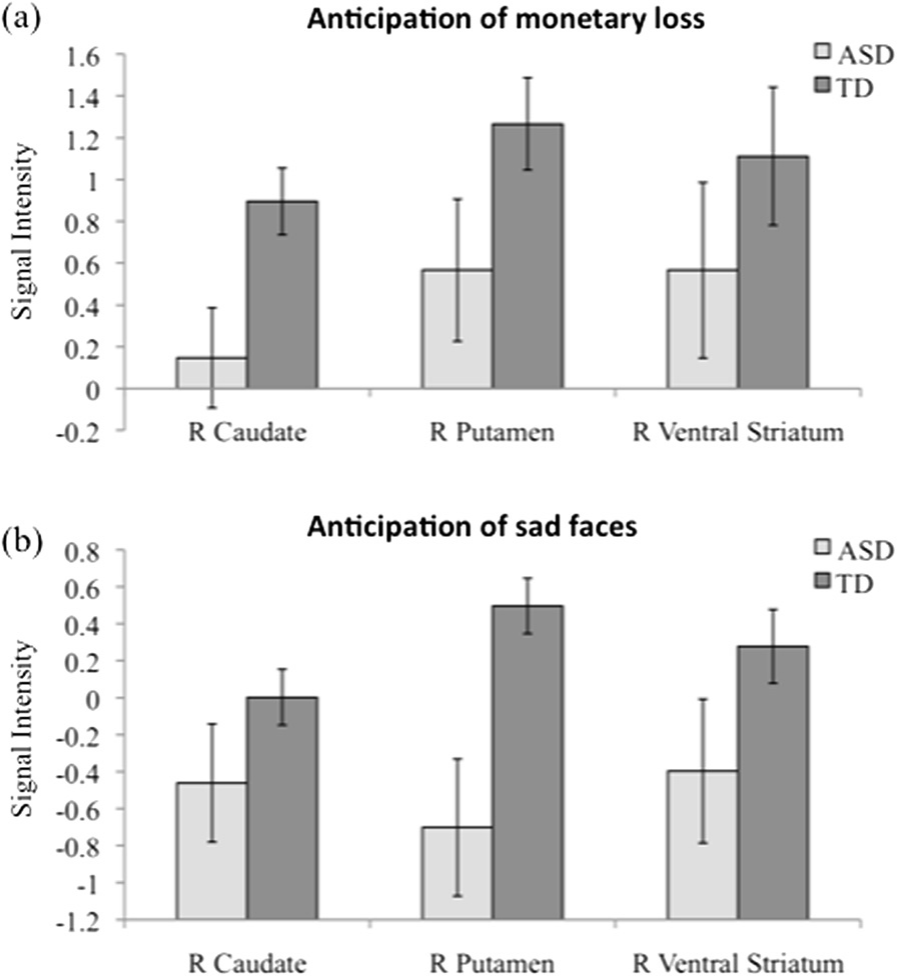
**Between-group differences in the social and non-social conditions using structural regions of interest (ROIs).** Structural ROI results demonstrated significantly reduced activation in the ASD group during the anticipation of **(a)** monetary loss and **(b)** sad faces.

A 2 (Group: ASD, TD) × 2 (Run Type: Social, Non-social) ANOVA was conducted for each of the six striatal regions of interest (right/left NAc, right/left caudate nucleus, and right/left putamen). No significant Group × Run Type interactions were found. A main effect of Run Type was found in all six regions with significantly greater activation in the non-social versus the social condition, all *P*’s < .002. A main effect of Group was found in the right caudate nucleus, *F*(1,43) = 6.91, *P* = .012, right putamen, *F*(1,43) = 8.03, *P* = .007, and right NAc, *F*(1,43) = 4.53, *P* = .039, reflecting significantly reduced activation in these ROIs for the ASD group when compared to the TD group (see Figure 5).

We used the Benjamini-Hochberg method [65] to control for multiple comparisons arising from repeated testing of the group effect across regions. This method controls for the false discovery rate without the penalty to statistical power of other methods. Using this approach, a significant main effect of group was detected in the right caudate nucleus, adjusted *P* = .042, and in the right putamen, adjusted *P* = .036. In addition, a marginally significant effect of group was detected in the right NAc, adjusted *P* = .078.

### Correlations with ASD symptoms

**Figure 6:**
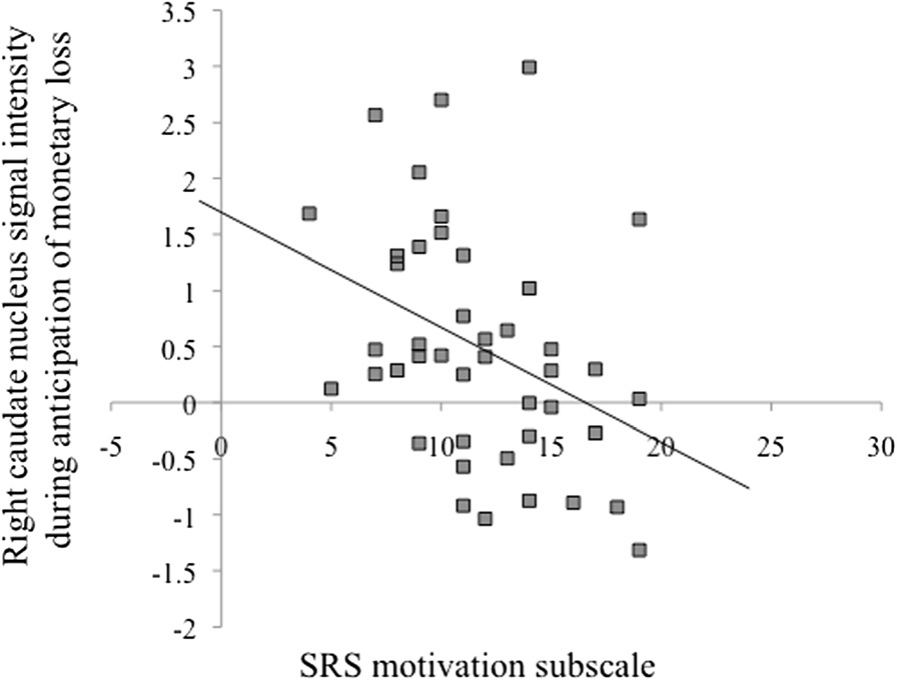
**Exploratory correlation between the SRS social motivation subscale and the magnitude of activation in the right caudate nucleus (structural ROI) during the anticipation of monetary loss across both groups.**

Exploratory correlations were examined among SRS scores (both total *t*-scores and social motivation subscale scores) and structural ROIs in the ventral and dorsal striatum (right and left NAc, right and left caudate nucleus, and right and left putamen) across both groups. No significant correlations were found among the structural ROIs and SRS total *t*-scores, all *P*’s > .05. A positive correlation was detected between the SRS social motivation subscale and the magnitude of activation in the right caudate nucleus while anticipating monetary loss, *r*(43) = −.37, *P* = .012 (see Figure 6). No significant correlations were detected between scores on the SRS social motivation subscale and the magnitude of activation in any structural ROIs while anticipating sad faces, all *P*’s > .05. We did not include correlational analyses between functional regions that differentiated groups and ASD symptoms because of the potential for non-independence between functional ROIs defined on the basis of group differences in activation and SRS scores that were correlated with group status^a^ [66].

## Discussion

The objective of the present study was to examine neural responses to negative social and non-social reinforcement in children and adolescents with ASD by using an incentive delay task. We found that children with ASD demonstrated reduced activation of the right caudate nucleus while anticipating monetary reward loss and reduced activation of several mesolimbic (amygdala, caudate nucleus, NAc, and putamen) and prefrontal cortical regions (ACC, frontal pole, inferior frontal gyrus, and insula) while anticipating sad faces presented in the context of a reward task. Across both groups, reduced activation of the right caudate nucleus while anticipating negative non-social reinforcement was negatively associated with SRS social motivation scores (that is, reduced responsivity to monetary loss cues was associated with greater impairment in social motivation).

This study extends the existing body of literature addressing the processing of social and non-social positive reinforcers in ASD and provides evidence that individuals with ASD may be hyporesponsive in canonical reward processing regions to the anticipation of potential social and non-social negative reinforcement. These findings complement previous studies of neural responses to reward gains in ASD, which have found attenuated activation of mesolimbic brain regions in response to social and monetary reward gains in ASD [8-11]. This study also supports previous research that the motivation to avoid failure or punishment is associated with different physiological responses in ASD and is differentially related to personality and clinical measures [13]. In addition, this study found evidence that the anticipation of negative social reinforcers is associated with hypoactivation of a more extensive network of frontostriatal regions than the anticipation of monetary reward loss in ASD, which corroborates several studies demonstrating greater deficits in social versus non-social reward processing in ASD [9,10].

When considered alongside the extant literature on reward processing in ASD, these results provide evidence that impaired social motivation in this population may involve attenuated responsivity to cues for both positive social reinforcement *and* negative social reinforcement. Reduced responsivity to negative social cues may contribute to the tendency of individuals with ASD to be less attuned to social exclusion [67] and thus less likely to engage in prosocial behaviors that build social relationships. This attenuated response to negative social reinforcement may be a particularly powerful factor in reduced social motivation since the motivation to avoid negative social reinforcement may be greater than the drive to seek out social rewards in individuals without ASD [46].

In particular, our finding of reduced NAc activation while anticipating a sad face suggests that negative social reinforcement may be less salient for individuals with ASD, given the critical role of the NAc in incentive salience [21,22]. Reduced activation of the dorsal striatum in the ASD group while anticipating negative social reinforcement is also notable given the role of the dorsal striatum in instrumental learning [30]. This finding suggests that children with ASD may be impaired in learning to anticipate negative social situations. For example, children with ASD may not learn which actions are associated with social rejection or how to apply this knowledge to modify their behavior. Accordingly, children with ASD may continue to act in a way that may be considered awkward or inappropriate by their peers even after experiencing negative social consequences for these behaviors - a conceptualization that is consistent with the literature indicating deficits in social reward learning in ASD [9,68].

The finding of reduced activation in both the ventral striatum and dorsal striatum for social negative reinforcement and reduced activation only in the dorsal striatum for non-social negative reinforcement is notable in light of models that delineate distinct functional roles for these regions in reward processing [30,69]. According to these models, the NAc serves as the ‘director’ or ‘critic’, which evaluates reward outcomes in order to learn how to predict future rewards. On the other hand, the dorsal striatum serves as the ‘actor,’ which codes for the action used to obtain a reward in order to guide future reward-related behavior. Although both the ventral striatum and dorsal striatum may code for motivational salience [21,22], the dorsal striatum in particular plays an important role in linking behavior to reward information [29,30] and is critical to instrumental learning [70]. The dorsal striatum is also critically involved in the development of habits, which are ritualized behaviors that are initiated based on positive and negative reinforcers yet persist even after reward devaluation or omission [12,71]. Therefore, hypoactivation of the dorsal striatum while anticipating negative social reinforcement may ultimately result in an inability to develop and maintain ritualized social behavior and other habitual behaviors in the absence of consistent reward feedback.

Atypical responding to cues predicting potential negative social reinforcement in ASD has important implications for reward learning, which occurs when there is a mismatch between an expected and an actual event. This mismatch is referred to as a prediction error [72-76], and it reflects the integration of information about current and predicted rewards. Learning occurs during both positive prediction errors (‘better than predicted’) and negative prediction errors (‘worse than predicted’) [77]. Therefore, atypical processing of cues for negative reinforcement would likely impact the ability to learn from prediction errors during reward feedback, and future studies that model outcome prediction errors will be needed to confirm the role of atypical striatal activation during outcome violations in the context of reward learning in ASD.

More broadly, the present findings provide further support that ASD may be characterized by domain-general dysfunction of brain reward circuitry. The present study, along with previous studies on this topic [8-11], have consistently found evidence for atypical reward circuit activation across a range of different study methodologies involving both reward loss and gain and both social and non-social stimuli. However, these domain-general findings raise the possibility that this atypicality may reflect a more basic neural dysfunction in ASD. For instance, ASD is characterized by atypical brain connectivity [78], including atypical connectivity of reward circuitry [79]. Thus, atypical reward circuitry activation may be a reflection of more widespread atypicalities of network connectivity in ASD. Alternatively, these results may be attributable to impairments in processing complex or abstract tasks as in the present study, as individuals with ASD may show atypicalities in processing complex stimuli [80,81] or in maintaining abstract internal representations [82,83].

The present study also found negative correlations between SRS social motivation scores and the magnitude of activation in the right caudate nucleus during monetary loss anticipation. These results indicate that the children and adolescents with ASD who were least sensitive to non-social negative reinforcers were characterized by greater impairments in social motivation. These findings highlight the clinical relevance of neural responses to negative reinforcers for social functioning in children and adolescents with ASD.

Limitations of the present study include the modest sample size, although it is commensurate with previous neuroimaging studies of reward processing in children with ASD [9,10]. Another limitation is the exploratory nature of the correlational analyses, which were not corrected for multiple comparisons. In addition, images of sad faces served as proxies for negative reinforcement in naturalistic social interactions - a complex and dynamic process that may involve facial expressions of anger, frustration, or social disapproval rather than sadness.

## Conclusions

The results of the present study suggest that children and adolescents with ASD show atypical neural responses to social and non-social negative reinforcement, with more extensive group differences in the social condition. Specifically, children and adolescents with ASD demonstrated hypoactivation in a network of frontostriatal regions (including the ventral striatum and dorsal striatum) when anticipating possible negative social reinforcement and hypoactivation of only the dorsal striatum when anticipating possible negative non-social reinforcement. Processing negative social reinforcement is critically important in driving the production of goal-directed social behaviors, motivating social learning processes, and maintaining social relationships. The study of reward system functioning in the context of negative social reinforcement may also have important clinical implications, as reward processing circuitry provides a novel target for the development of more effective behavioral and psychopharmacological interventions in these populations. Although many empirically supported interventions for ASD focus on enhancing responsivity to social interactions through positive social engagement [84,85], these interventions may be augmented by a therapy component that helps individuals with ASD to respond more effectively to negative social reinforcement (for example, facilitating the ability to learn from unsuccessful social bids). Future research will be needed to investigate whether interventions aimed at improving social motivation in ASD should target skills related to processing negative social reinforcers as a means to improved social skills and ultimately improved social functioning in ASD. Reward processing circuitry is known to undergo significant developmental changes in adolescence [86,87], and thus, it will be critical for future studies with larger samples to examine the developmental course of neural responses to negative social reinforcement in individuals with and without ASD.

## Endnote

^a^We thank an anonymous reviewer for raising this point.

## References

[1] APA (2013). Diagnostic and statistical manual of mental disorders: DSM-V.

[2] Dawson G, Toth K, Abbott R, Osterling J, Munson J, Estes A (2004). Early social attention impairments in autism: social orienting, joint attention, and attention to distress. Dev Psychol.

[3] Chevallier C, Kohls G, Troiani V, Brodkin ES, Schultz RT (2012). The social motivation theory of autism. Trends Cogn Sci.

[4] Chevallier C, Grèzes J, Molesworth C, Berthoz S, Happé F (2011). Brief report: selective social anhedonia in high functioning autism. J Autism Dev Disord.

[5] Soderstrom H, Rastam M, Gillberg C (2002). Temperament and character in adults with Asperger syndrome. Autism.

[6] Chawarska K, Volkmar F, Klin A (2010). Limited attentional bias for faces in toddlers with autism spectrum disorders. Arch Gen Psychiatry.

[7] Dichter GS, Damiano CR, Allen JA (2012). Reward circuitry dysfunction in psychiatric and neurodevelopmental disorders and genetic syndromes: animal models and clinical findings. J Neurodev Dis.

[8] Dichter GS, Richey JA, Rittenberg AM, Sabatino A, Bodfish JW (2012). Reward circuitry function in autism during face anticipation and outcomes. J Autism Dev Disord.

[9] Scott-Van Zeeland AA, Dapretto M, Ghahremani DG, Poldrack RA, Bookheimer SY (2010). Reward processing in autism. Autism Res.

[10] Delmonte S, Balsters JH, McGrath J, Fitzgerald J, Brennan S, Fagan AJ (2012). Social and monetary reward processing in autism spectrum disorders. Mol Autism.

[11] Dichter GS, Felder JN, Green SR, Rittenberg AM, Sasson NJ, Bodfish JW (2012). Reward circuitry function in autism spectrum disorders. Soc Cogn Affect Neurosci.

[12] Everitt BJ, Robbins TW (2005). Neural systems of reinforcement for drug addiction: from actions to habits to compulsion. Nat Neurosci.

[13] South M, Dana J, White SE, Crowley MJ (2011). Failure is not an option: risk-taking is moderated by anxiety and also by cognitive ability in children and adolescents diagnosed with an autism spectrum disorder. J Autism Dev Disord.

[14] Larson MJ, South M, Krauskopf E, Clawson A, Crowley MJ (2011). Feedback and reward processing in high-functioning autism. Psychiatry Res.

[15] Gray JA, Kumari V, Lawrence N, Young AM (1999). Functions of the dopaminergic innervation of the nucleus accumbens. Psychobiology.

[16] Young PT (1959). The role of affective processes in learning and motivation. Psychol Rev.

[17] Horvitz J (2000). Mesolimbocortical and nigrostriatal dopamine responses to salient non-reward events. Neuroscience.

[18] Salamone JD (1994). The involvement of nucleus accumbens dopamine in appetitive and aversive motivation. Behav Brain Res.

[19] Delgado MR, Nystrom LE, Fissell C, Noll D, Fiez JA (2000). Tracking the hemodynamic responses to reward and punishment in the striatum. J Neurophysiol.

[20] Tom SM, Fox CR, Trepel C, Poldrack RA (2007). The neural basis of loss aversion in decision-making under risk. Science.

[21] Cooper JC, Knutson B (2008). Valence and salience contribute to nucleus accumbens activation. Neuroimage.

[22] Carter RM, MacInnes JJ, Huettel SA, Adcock RA (2009). Activation in the VTA and nucleus accumbens increases in anticipation of both gains and losses. Front Behav Neurol.

[23] Delgado MR, Jou RL, LeDoux JE, Phelps EA (2009). Avoiding negative outcomes: tracking the mechanisms of avoidance learning in humans during fear conditioning. Frontiers Behav Neurol.

[24] Jensen J, McIntosh AR, Crawley AP, Mikulis DJ, Remington G, Kapur S (2003). Direct activation of the ventral striatum in anticipation of aversive stimuli. Neuron.

[25] Seymour B, Daw N, Dayan P, Singer T, Dolan R (2007). Differential encoding of losses and gains in the human striatum. J Neurosci.

[26] Knutson B, Adams CM, Fong GW, Hommer D (2001). Anticipation of increasing monetary reward selectively recruits nucleus accumbens. J Neurosci.

[27] Elliott R, Friston KJ, Dolan RJ (2000). Dissociable neural responses in human reward systems. J Neurosci.

[28] Knutson B, Westdorp A, Kaiser E, Hommer D (2000). FMRI visualization of brain activity during a monetary incentive delay task. Neuroimage.

[29] Delgado MR, Miller MM, Inati S, Phelps EA (2005). An fMRI study of reward-related probability learning. Neuroimage.

[30] O’Doherty J, Dayan P, Schultz J, Deichmann R, Friston K, Dolan RJ (2004). Dissociable roles of ventral and dorsal striatum in instrumental conditioning. Science.

[31] O’Doherty J, Critchley H, Deichmann R, Dolan RJ (2003). Dissociating valence of outcome from behavioral control in human orbital and ventral prefrontal cortices. J Neurosci.

[32] Knutson B, Greer SM (2008). Anticipatory affect: neural correlates and consequences for choice. Philos Trans R Soc B Sci.

[33] Simmons A, Matthews SC, Stein MB, Paulus MP (2004). Anticipation of emotionally aversive visual stimuli activates right insula. Neuroreport.

[34] Preuschoff K, Quartz SR, Bossaerts P (2008). Human insula activation reflects risk prediction errors as well as risk. J Neurosci.

[35] Kim H, Shimojo S, O’Doherty JP (2006). Is avoiding an aversive outcome rewarding? Neural substrates of avoidance learning in the human brain. PLoS Biol.

[36] Schoenbaum G, Chiba AA, Gallagher M (1998). Orbitofrontal cortex and basolateral amygdala encode expected outcomes during learning. Nat Neurosci.

[37] Schoenbaum G, Esber GR (2010). How do you (estimate you will) like them apples? Integration as a defining trait of orbitofrontal function. Curr Opin Neurobiol.

[38] Tremblay L, Schultz W (2000). Modifications of reward expectation-related neuronal activity during learning in primate orbitofrontal cortex. J Neurophysiol.

[39] Takahashi YK, Roesch MR, Wilson RC, Toreson K, O’Donnell P, Niv Y (2011). Expectancy-related changes in firing of dopamine neurons depend on orbitofrontal cortex. Nat Neurosci.

[40] Amiez C, Joseph JP, Procyk E (2006). Reward encoding in the monkey anterior cingulate cortex. Cereb Cortex.

[41] Amiez C, Joseph JP, Procyk E (2005). Anterior cingulate error‐related activity is modulated by predicted reward. Eur J Neurosci.

[42] Yacubian J, Gläscher J, Schroeder K, Sommer T, Braus DF, Büchel C (2006). Dissociable systems for gain-and loss-related value predictions and errors of prediction in the human brain. J Neurosci.

[43] Hamann SB, Ely TD, Hoffman JM, Kilts CD (2002). Ecstasy and agony: activation of the human amygdala in positive and negative emotion. Psychol Sci.

[44] Baxter MG, Murray EA (2002). The amygdala and reward. Nat Rev Neurosci.

[45] Balliet D, Mulder LB, Van Lange PA (2011). Reward, punishment, and cooperation: a meta-analysis. Psychol Bull.

[46] Kohls G, Perino MT, Taylor JM, Madva EN, Cayless SJ, Troiani V (2013). The nucleus accumbens is involved in both the pursuit of social reward and the avoidance of social punishment. Neuropsychologia.

[47] Kaufman AS, Kaufman NL (1990). Kaufman brief intelligence test- second edition.

[48] Lord C, Risi S, Lambrecht L, Cook EH, Leventhal BL, DiLavore PC (2000). The autism diagnostic observation schedule - generic: a standard measure of social and communication deficits associated with the spectrum of autism. J Autism Dev Disord.

[49] Gotham K, Pickles A, Lord C (2009). Standardizing ADOS scores for a measure of severity in autism spectrum disorders. J Autism Dev Disord.

[50] Hus V, Lord C (2014). The autism diagnostic observation schedule, module 4: revised algorithm and standardized severity scores. J Autism Dev Disord.

[51] Tottenham N, Tanaka JW, Leon AC, McCarry T, Nurse M, Hare TA (2009). The NimStim set of facial expressions: Judgments from untrained research participants. Psychiatry Res.

[52] Smith SM (2002). Fast robust automated brain extraction. Hum Brain Mapp.

[53] Jenkinson M, Bannister P, Brady M, Smith SM (2002). Improved optimization for the robust and accurate linear registration and motion correction of brain images. Neuroimage.

[54] Smith SM, Jenkinson M, Woolrich MW, Beckmann CF, Behrens TEJ, Johansen-Berg H (2004). Advances in functional and structural MR image analysis and implementation as FSL. Neuroimage.

[55] Jenkinson M, Smith SM (2001). A global optimisation method for robust affine registration of brain images. Med Image Anal.

[56] Wilke M, Schmithorst VJ, Holland SK (2002). Assessment of spatial normalization of whole-brain magnetic resonance images in children. Hum Brain Mapp.

[57] Burgund ED, Kang HC, Kelly JE, Buckner RL, Snyder AZ, Petersen SE (2002). The feasibility of a common stereotactic space for children and adults in fMRI studies of development. Neuroimage.

[58] Uddin LQ, Davies MS, Scott AA, Zaidel E, Bookheimer SY, Iacoboni M (2008). Neural basis of self and other representation in autism: an FMRI study of self-face recognition. PLoS One.

[59] Bookheimer SY, Wang A, Scott A, Sigman M, Dapretto M (2008). Frontal contributions to face processing differences in autism: evidence from fMRI of inverted face processing. J Int Neuropsychol Soc.

[60] Woolrich MW, Ripley BD, Brady M, Smith SM (2001). Temporal autocorrelation in univariate linear modeling of FMRI data. Neuroimage.

[61] Beckmann CF, Jenkinson M, Smith SM (2003). General multilevel linear modeling for group analysis in FMRI. Neuroimage.

[62] Constantino JN, Gruber CP (2002). The social responsiveness scale.

[63] White SW, Koenig K, Scahill L (2010). Group social skills instruction for adolescents with high-functioning autism spectrum disorders. Focus Autism Dev Disabil.

[64] Scheeren AM, Koot HM, Begeer S (2012). Social interaction style of children and adolescents with high-functioning autism spectrum disorder. J Autism Dev Disord.

[65] Benjamini Y, Hochberg Y (1995). Controlling the false discovery rate: a practical and powerful approach to multiple testing. J Royal Stat Soc Series B (Methodological).

[66] Vul E, Harris C, Winkielman P, Pashler H (2009). Puzzlingly high correlations in fMRI studies of emotion, personality, and social cognition. Perspect Psychol Sci.

[67] McPartland JC, Crowley MJ, Perszyk DR, Naples AJ, Mukerji CE, Wu J (2011). Temporal dynamics reveal atypical brain response to social exclusion in autism. Dev Cogn Neurosci.

[68] Lin A, Adolphs R, Rangel A (2012). Impaired learning of social compared to monetary rewards in autism. Front Neurosci.

[69] Atallah HE, Lopez-Paniagua D, Rudy JW, O’Reilly RC (2006). Separate neural substrates for skill learning and performance in the ventral and dorsal striatum. Nat Neurosci.

[70] Wächter T, Lungu OV, Liu T, Willingham DT, Ashe J (2009). Differential effect of reward and punishment on procedural learning. J Neurosci.

[71] Graybiel AM (2008). Habits, rituals, and the evaluative brain. Annu Rev Neurosci.

[72] McClure SM, Daw ND, Read MP (2003). A computational substrate for incentive salience. Trends Neurosci.

[73] Schultz W, Dayan P, Montague PR (1997). A neural substrate of prediction and reward. Science.

[74] Schultz W, Dickinson A (2000). Neuronal coding of prediction errors. Annu Rev Neurosci.

[75] Seymour B, O’Doherty JP, Dayan P, Koltzenburg M, Jones AK, Dolan RJ (2004). Temporal difference models describe higher-order learning in humans. Nature.

[76] Hikosaka O, Nakamura K, Nakahara H (2006). Basal ganglia orient eyes to reward. J Neurophysiol.

[77] Schultz W (1998). Predictive reward signal of dopamine neurons. J Neurophysiol.

[78] Belmonte MK, Allen G, Beckel-Mitchener A, Boulanger LM, Carper RA, Webb SJ (2004). Autism and abnormal development of brain connectivity. J Neurosci.

[79] Di Martino A, Kelly C, Grzadzinski R, Zuo XN, Mennes M, Mairena MA (2010). Aberrant striatal functional connectivity in children with autism. Biol Psychiatry.

[80] Minshew NJ, Goldstein G (1998). Autism as a disorder of complex information processing. Ment Retard Dev Disabil Res Rev.

[81] Williams DL, Goldstein G, Minshew NJ (2006). Neuropsychologic functioning in children with autism: further evidence for disordered complex information-processing. Child Neuropsychol.

[82] Gastgeb HZ, Rump KM, Best CA, Minshew NJ, Strauss MS (2009). Prototype formation in autism: can individuals with autism abstract facial prototypes?. Autism Res.

[83] Ropar D, Peebles D (2007). Sorting preference in children with autism: the dominance of concrete features. J Autism Dev Disord.

[84] Rogers SJ (2000). Interventions that facilitate socialization in children with autism. J Autism Dev Disord.

[85] McConnell SR (2002). Interventions to facilitate social interaction for young children with autism: review of available research and recommendations for educational intervention and future research. J Autism Dev Disord.

[86] Eshel N, Nelson EE, Blair RJ, Pine DS, Ernst M (2007). Neural substrates of choice selection in adults and adolescents: development of the ventrolateral prefrontal and anterior cingulate cortices. Neuropsychologia.

[87] Galvan A, Hare TA, Parra CE, Penn J, Voss H, Glover G (2006). Earlier development of the accumbens relative to orbitofrontal cortex might underlie risk-taking behavior in adolescents. J Neurosci.

